# Potential Anticancer Activity of Donkey Milk in Human Gastric Adenocarcinoma (AGS) Cell Line

**DOI:** 10.3390/biology15151279

**Published:** 2026-08-04

**Authors:** Mariangela Mazzone, Maria Carmela Di Marcantonio, Maria Sindaco, Antonella Fatica, Noemi Mencarelli, Marialucia Gallorini, Amelia Cataldi, Raffaella Muraro, Elisabetta Salimei, Gabriella Mincione

**Affiliations:** 1 Department of Innovative Technologies in Medicine & Dentistry, University “G. d’Annunzio” Chieti-Pescara, 66100 Chieti, Italy; mariangelamazzone84@gmail.com (M.M.); dimarcantonio@unich.it (M.C.D.M.); mari.sindaco@gmail.com (M.S.); raffaella.muraro@unich.it (R.M.); 2 Department of Agricultural, Environmental and Food Sciences, University of Molise, 86100 Campobasso, Italy; antonella.fatica@unimol.it (A.F.); salimei@unimol.it (E.S.); 3 Department of Pharmacy, University “G. d’Annunzio” Chieti-Pescara, 66100 Chieti, Italy; noemi.mencarelli@phd.unich.it (N.M.); marialucia.gallorini@unich.it (M.G.); amelia.cataldi@unich.it (A.C.)

**Keywords:** donkey milk, gastric cancer, adjunctive therapy, nutraceutical agent

## Abstract

Gastric cancer (GC) remains a major global health challenge, ranking among the most lethal malignancies due to late diagnosis, high tumor heterogeneity, and limited treatment efficacy. The search for safer, nutritionally based adjuncts to conventional therapies is therefore a research priority. Donkey milk (DM), traditionally used as a hypoallergenic substitute for infants, is emerging as a functional food with remarkable bioactivity. Its composition closely resembles human milk, with high levels of bioactive proteins, a favorable polyunsaturated lipid profile, antioxidant vitamins, and immune-supportive minerals. Despite its growing nutraceutical appeal, the anticancer potential of DM in GC has not yet been explored. This study represents the first investigation of DM in human gastric adenocarcinoma (AGS) cells. Using increasing concentrations of whole DM (25–100%), a dose-dependent inhibition of cell viability and migration was observed. Mechanistic insights reveal that DM induces mitochondrial oxidative stress, disrupts cell cycle progression (S/G2 accumulation at 75%, G2 arrest at 100%), and unexpectedly triggers a pro-inflammatory gene signature suggesting stress-driven immunostimulation rather than canonical apoptosis. These findings highlight a non-classical, context-dependent cytotoxic mechanism that distinguishes DM from conventional pro-apoptotic agents. DM may represent a promising nutraceutical candidate for GC management, bridging traditional food resources with modern oncology. By inhibiting hallmark cancer traits while engaging unique immunological pathways, DM offers a sustainable, low-toxicity approach with translational potential. Future studies will focus on the characterization of active components, validation in organoid and animal models, and exploring clinical applications of DM-derived bioactive components in cancer prevention and therapy.

## Simple Summary

Stomach cancer is a major health challenge because it is often diagnosed late and can be resistant to standard drugs. This study explored a natural alternative: donkey milk. Already known for being hypoallergenic and rich in immune-boosting nutrients, this is the first time its effects have been tested on human gastric cancer cells. The research highlights three main findings: (1) inhibition of growth—high concentrations of the milk significantly stop cancer cells from proliferating (rapidly multiplying); (2) blocking spread—it prevents migration, which is the ability of cancer cells to move and invade other tissues; and (3) low toxicity—unlike harsh chemotherapy, donkey milk works by creating “internal stress” that disrupts the cancer cells’ life cycle without being highly toxic to the body. By activating specific signals linked to inflammation, donkey milk shows a unique ability to suppress tumors naturally. While these laboratory results are promising, more research is needed to confirm if these benefits work the same way in human patients. Ultimately, donkey milk could become a valuable, gentle add-on therapy in cancer care.

## 1. Introduction

Gastric cancer (GC) remains a significant global health concern, with notable disparities in incidence and outcomes between regions. Globally, it ranks as the fifth-most diagnosed and fourth-deadliest cancer. Adenocarcinoma is the most common histological subtype, accounting for about 95% of malignant stomach tumors. Ethnic disparities are evident, with higher rates among Asian/Pacific Islander, Black, Indigenous/Alaska Native, and Hispanic populations in the U.S. compared to White populations [1]. The disease shows a strong male predominance (2:1 ratio) and is rare in individuals under 45, but African patients tend to be younger and have a higher proportion of women affected compared to other regions [1]. Most patients are diagnosed at advanced or metastatic stages, leading to poor 5-year survival (7% for metastatic, ~75% for localized disease) [2,3,4]. Despite advances in diagnostics, molecular subtyping, and targeted therapies, tumor heterogeneity and the complexity of the tumor microenvironment continue to hinder therapeutic success [5,6,7]. Preventive measures such as *Helicobacter pylori* (*H. pylori*) eradication and screening programs have improved early detection and outcomes, but systemic therapies remain central to management for most patients [8,9,10].

In this challenging clinical context, there is growing interest in adjunctive, non-toxic, and nutritionally based therapies for gastrointestinal malignancies. Comprehensive evidence indicates that dietary bioactive components, including phytochemicals, functional food concentrates, and specific milk derivates, exert significant protective effects against gastric by inducing apoptosis, inhibiting angiogenesis, and modulating the gastric microbiota [11,12]. Donkey milk (DM) has emerged as a promising functional food due to its unique composition and promising health benefits, closely resembling human milk (HM) in protein profile and bioactive compounds, which underpins its established use as a hypoallergenic alternative in infants with cow milk protein allergy [13,14,15,16].

**Figure 1:**
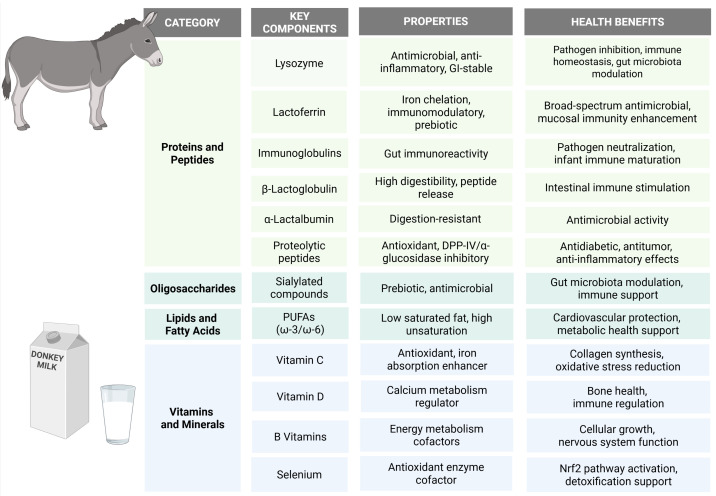
Bioactive compounds in DM and their properties. The figure illustrates the key functional components of DM, including highly digestible and low-allergenic proteins and peptides (lysozyme, lactoferrin, immunoglobulins, β-lactoglobulin, α-lactalbumin, and proteolytic peptides) with antimicrobial and immunomodulatory effects; oligosaccharides (sialylated compounds) for microbiota and immune support; and a cardioprotective lipid profile rich in polyunsaturated fatty acids (PUFAs) and essential micronutrients (vitamins C, D, B complex, and selenium) contributing to antioxidant defenses and immune function (created with Biorender.com).

Characterized by a low fat content and a favorable lipid profile rich in polyunsaturated fatty acids (PUFAs), DM exhibits cardioprotective potential and enhanced digestibility comparable to HM (Figure 1) [17,18,19,20,21,22]. Its protein fraction presents a balanced casein-to-whey ratio, with high digestibility and low allergenicity, highlighted by abundant bioactive proteins such as lysozyme, lactoferrin, and immunoglobulins, which contribute to antimicrobial, antioxidant, and immunomodulatory effects (Figure 1) [14,17,18,19,22,23,24,25,26,27,28,29,30]. Additionally, DM contains high lactose levels that support calcium absorption and gut microbiota development [17,18,19,23]. It is also rich in vitamins C, D, and B complex and minerals such as selenium, which enhance antioxidant capacity and immune function (Figure 1) [22,30,31]. Beyond the action of individual bioactive components, recent research has focused on milk extracellular vesicles (MEVs). Donkey-derived MEVs, structurally similar to those in HM, exhibit pronounced anti-inflammatory effects and hold promise as natural nanocarriers for targeted nutraceutical and therapeutic applications in chronic inflammatory diseases [32,33]. Previous investigations have primarily focused on the cytotoxic and anti-proliferative effects of DM and its whey protein fractions against specific malignancies, such as breast, lung, and colorectal cancer models, often attributing these properties to the induction of apoptosis, upregulation of protective cytokines, or the synergistic action of lysozyme and lactoferrin. However, these antineoplastic responses have been shown to be strictly cancer type-, dose-, and processing-dependent. Despite the well-documented susceptibility of gastrointestinal tracts to dietary bioactive compounds, no studies to date have explored the anticancer or anti-migratory activity of DM in gastric cancer models, even though emerging research continues to explore its broader anticancer potential [34,35,36].

In response to the urgent need for safer adjunctive therapies in GC, this in vitro investigation, focused on key cancer hallmarks, aimed to explore for the first time the anticancer potential of DM in human gastric adenocarcinoma (AGS) cells. Through systematically comparing different concentrations of DM, this work uniquely aimed to bridge the gap between traditional food resources and modern oncology, introducing a novel nutraceutical approach that bridges traditional food resources with modern biomedical research. We ensured the sustainable sourcing and traceability of biological materials by establishing a collaboration with a commercial donkey farm. This allowed us to integrate traditional agriculture with cutting-edge biomedical research. Our in vitro investigation focused on key cancer hallmarks—including proliferation, migration, oxidative stress, and gene expression related to inflammation and apoptosis—with the aim of exploring the potential of DM as a nutraceutical agent in GC management.

## 2. Materials and Methods

### 2.1. Milk Sampling and Chemical Analysis

In the present study, donkey milk samples were obtained from 31 clinically healthy pluripara jennies at 135.5 (±60.1) days of lactation. Donkeys were raised in a commercial dairy farm (Ciucolandia), located at about 400 m of altitude (Capestrano, AQ, Italy; 42°17′12.2″ N 13°45′44.7″ E). The jennies had continuous access to water and alfalfa hay, and were supplemented daily with a 1.5 kg concentrate of corn and oats in equal proportions. Dams and foals live together and are separated three hours before the mechanical milking session (11:00 a.m.), as reported in the literature [37]. Individual milk was pooled according to the stage of lactation (A = early lactation 30–90 d; B = intermediate lactation 91–180 d; C = late lactation > 181 d) and sampled. Bulk milk was sampled. All samples were immediately transported under refrigerated conditions at 4 °C to the laboratory, and bulk milk samples were centrifuged at 1000× *g* for 20 min at 4 °C to remove the fat fraction. A, B, and C milk and bulk milk samples were divided in two fractions (centrifuged and non-centrifuged). Each fraction was then divided into three aliquots. The processing was completed within 24 h of collection to minimize any potential degradation of the milk components, as per Fantuz et al. [38]. DM samples (n = 8) were analyzed in triplicate for chemical components, including total solids, crude protein, fats, urea, and ash [39].

### 2.2. Cell Cultures and Treatments

The AGS human gastric adenocarcinoma cell line (Cell Lines Services, Eppelheim, Germany) was cultured as previously described [40]. Briefly, the AGS cells were cultured in Dulbecco’s modified Eagle’s medium (DMEM) supplemented with 4.5 g/L glucose, 2 mM L-glutamine, and 10% fetal bovine serum. For the experimental assays, cells were exposed to increasing concentrations of DM diluted in complete culture medium: 0% (untreated cells used as negative control), 25%, 50%, 75%, and 100% DM. The concentrations of DM used in this study were selected based on previous studies on milk-derived matrices. Furthermore, as this study represents a preliminary step toward in vivo or dietary intervention studies, the experimental designed involved testing DM in its raw, unprocessed form. This approach allows closer simulation of a direct, high-dose exposure scenario at the gastric level. Exposure duration was adjusted according to the specific experimental endpoints. All cell-based experiments were performed in triplicate (n = 3).

### 2.3. Wound Healing Assay

AGS cells were seeded in a 12-well plate at a density of 2 × 10^5^ cells per well. When the cells reached a nearly confluent state, a wound was created using a sterile 10 μL pipette tip. After gentle washing with phosphate-buffered saline, the cells were treated with fresh DMEM containing different concentrations of DM (as mentioned above). The progress of cell migration near the crossing point was captured at 0 and 24 h post-wounding under an inverted microscope for cell culture (DMi1, Leica Microsystems Srl, Buccinasco, MI, Italy). Images were analyzed using ImageJ 1.54t.

### 2.4. Crystal Violet Assay and Morphological Analysis

AGS cells, cultured and treated as previously described, were fixed with 4% glutaraldehyde and stained with crystal violet at the established time points (24 and 48 h). Images were captured using a light phase-contrast microscope equipped with a camera. The stained cells were washed with 70% (*v*/*v*) ethanol under constant agitation for 2 h to solubilize crystal violet. The absorbance of the extracted dye was measured at 570 nm using a spectrophotometer (Thermo Fisher Scientific, Waltham, MA, USA).

### 2.5. Lactate Dehydrogenase Assay

To evaluate the cytotoxic effects of DM on AGS cells, lactate dehydrogenase (LDH) release into the culture medium was measured using a CytoTox 96^®^ non-radioactive cytotoxicity assay (Promega, Madison, WI, USA) following the manufacturer’s instructions [41]. Absorbance was measured at 490 and 690 nm (background) using a Multiskan GO spectrophotometer (Thermo Fisher Scientific, Waltham, MA, USA).

### 2.6. Detection of Mitochondrial Superoxide Anions

AGS cells were cultured and treated as previously described. The formation of reactive oxygen species (specifically mitochondrial superoxide anion) was detected by flow cytometry. Cells were stained with the fluorescent dye MitoSOX™ Red (Thermo Fisher, Waltham, MA, USA) at a concentration of 1 µL/mL (stock solution 1 mM in DMSO), and fluorescence was measured using a Beckman Coulter CytoFLEX flow cytometer (Brea, CA, USA). Data were analyzed with CytExpert Software version 1.2 (Beckman Coulter, Brea, CA, USA), and the results are expressed as mean fluorescence intensity (MFI).

### 2.7. Cell Cycle Analysis

Approximately 0.5 × 10^6^ cells per experimental condition (75% and 100% DM in complete medium) were harvested, fixed in 70% cold ethanol, and stored at 4 °C overnight. Cells were then suspended in 20 μg/mL propidium iodide (PI) and 100 μg/mL RNAse, final concentration. PI fluorescence was detected by a flow cytometer equipped with a 488 nm laser CytoFlex flow cytometer (Beckman Coulter, Brea, CA, USA) in the FL-3 channel. A minimum of 10,000 events/sample were acquired and analyzed with CytExpert software, version 1.2 (Beckman Coulter, Brea, CA, USA), and the percentages of cells in the G1, S, or G2 phase of the cell cycle were calculated using ModFit LT™ software, version 5.0 (Verity Software House, Topsham, ME, USA).

### 2.8. RNA Extraction and Quantitative Real-Time RT-PCR

Total RNA was extracted from untreated AGS cells and from AGS cells treated for 24 h with 75% DM and 100% DM in complete medium using Trifast (EuroClone S.p.A., Pero, Milan, Italy), in agreement with the manufacturer’s protocol. RNA samples were assessed for purity and quantified by a Nanodrop 1000 spectrophotometer (Applied Biosystems, Thermo Fisher Scientific, Waltham, MA, USA). Targets and reference genes were amplified in a volume of 10 μL containing 1 μL template cDNA, 0.2 μL of primer mixture and 5 μL of GoTaq^®^ 2-Step RT-qPCR System (Promega) according to the manufacturer’s instructions. cDNA levels were evaluated using IDT primers (Integrated DNA Technologies, Leuven, Belgium) by SYBR green quantitative real-time RT-PCR (qRT-PCR) analysis using the StepOneTM 2.0 Real-Time PCR system (Applied Biosystems). The cycling conditions were performed as follows: 10 min at 95 °C and 40 cycles of 15 s at 95 °C, followed by 1 min at 60 °C, and final elongation of 15 s at 95 °C. Data were analyzed using the comparative Ct method and were graphically indicated as 2^−∆∆Ct^ ± SD. cDNA amounts of the target genes were normalized by the ratio on the median value of the endogenous housekeeping gene β-actin, obtained in each treated cell vs. untreated cell.

For each experimental condition, expression of target genes (Table 1) was assessed in three replicates across three independent experiments.

### 2.9. Statistical Analysis

All data were subjected to descriptive statistical analysis using Microsoft Excel ProPlus. Inferential statistical analysis was performed with GraphPad Prism 8.0. For each treatment group, data are expressed as means ± standard deviation (SD). Differences between groups were assessed by one-way analysis of variance (ANOVA), followed by Dunnett’s and Tukey’s multiple comparison tests. Statistical significance was accepted at *p* < 0.05.

## 3. Results

### 3.1. Chemical Composition

**Figure 2:**
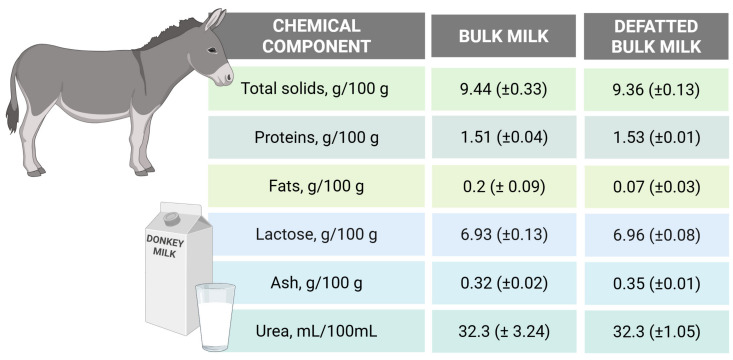
Chemical components of whole and defatted bulk DM samples (n = 8; mean values ± standard deviation) (created with Biorender.com).

**Figure 3:**
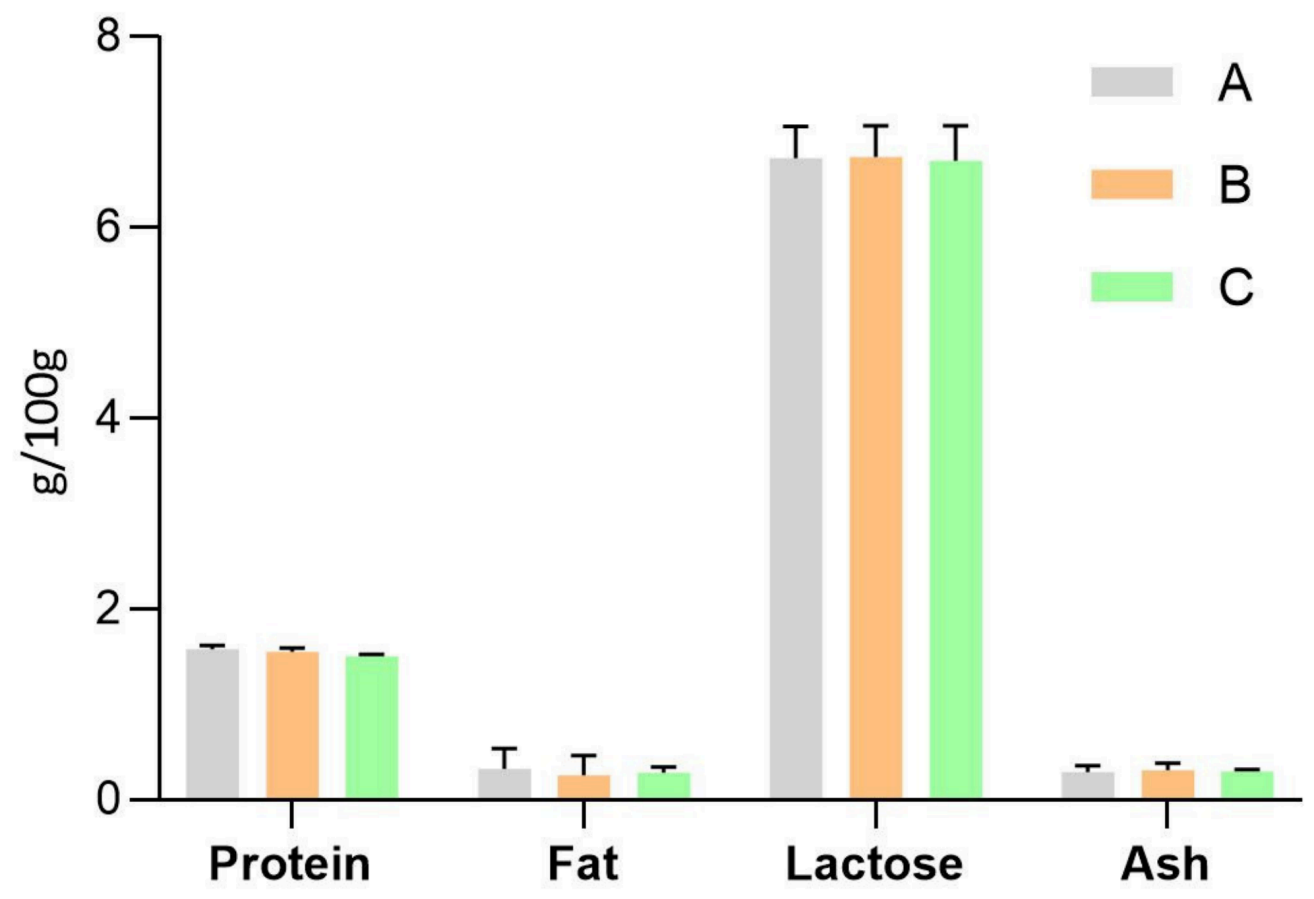
Chemical composition of DM at different stages of lactation (mean values ± standard deviation). A = early (30–90 d), B = mid (91–180 d), C = late (>181 d).

To assess lactation-stage variability, DM samples from three distinct stages of lactation (A = early lactation 30–90 d; B = intermediate lactation 91–180 d; C = late lactation >181 d) underwent comparative statistical analysis. Figure 2 details the chemical composition of bulk milk samples used in the present study. The results showed that the average total solid content of DM ranged from 9.04 (±0.64, SD) g/100 g to 9.16 (±0.40) g/100 g, observed in late lactation (C) and in early lactation (A), respectively. The same stability was observed for chemical components (Figure 2). DM urea content averaged 29.30 (±4.9) mL/100 mL, 29.56 (±4.6) mL/100 mL, and 28.32 (±6.91) mL/100 mL, in early, intermediate, and late lactation, respectively. As better depicted by Figure 3, no statistically significant differences (*p* > 0.05) were detected across lactation stages for any quantified parameter. This compositional homogeneity justified the use of defatted pooled bulk milk for all subsequent cellular assays, as it effectively precluded lactation-stage variability as a confounding factor.

### 3.2. Wound Closure Quantification

**Figure 4:**
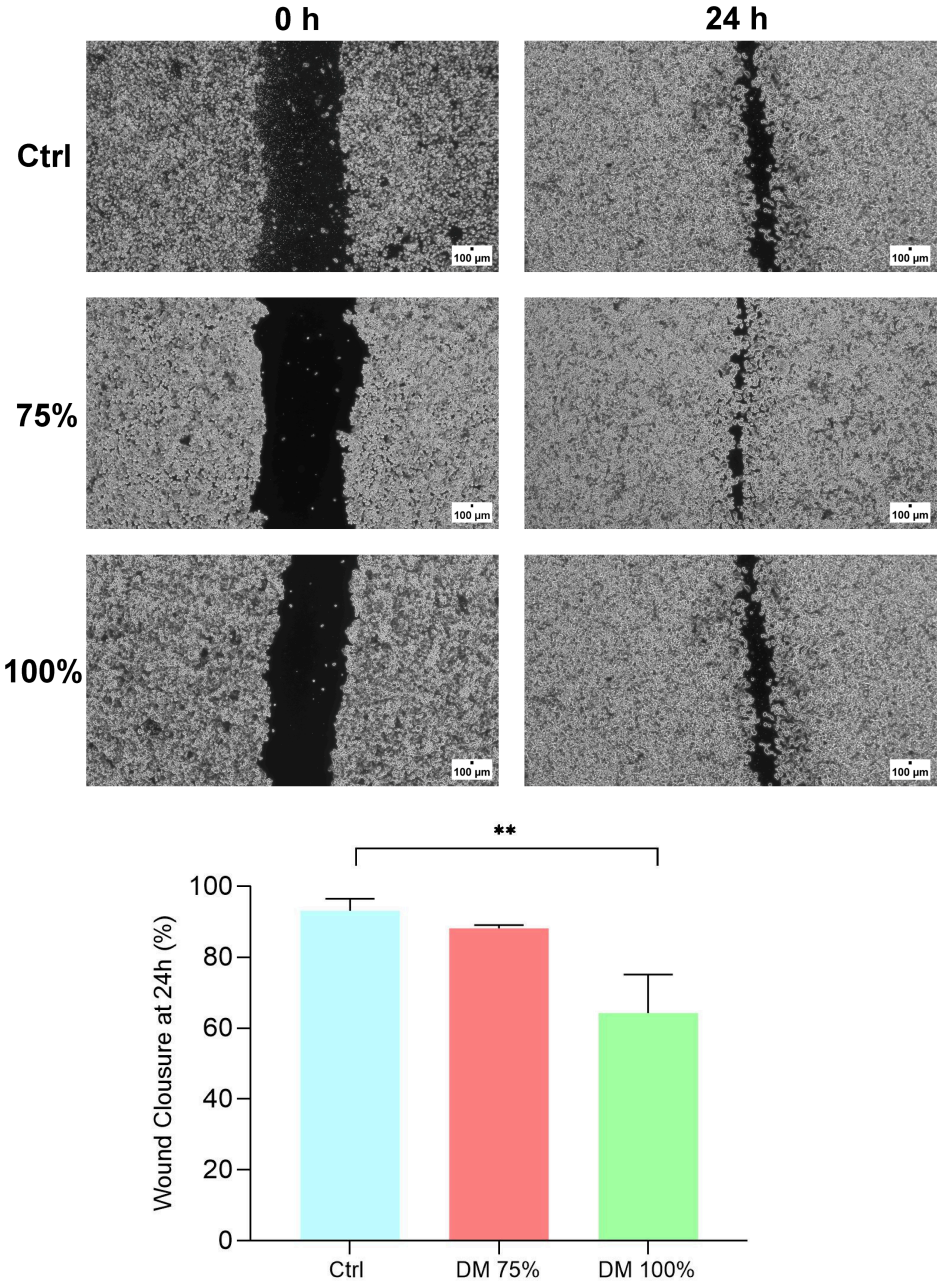
Effects of donkey milk (DM) on AGS cell migration assessed by wound healing assay. Representative images (10× magnification; scale bar = 100 μm) were acquired immediately after scratching (0 h) and after 24 h in untreated cells (CTRL) or cells treated with 75% or 100% DM in complete medium, using a Leica DMi1 inverted microscope. Percentage of wound closure at 24 h, calculated as [(wound area at 0 h − wound area at 24 h)/wound area at 0 h] × 100. Data are presented as means ± SD from three independent experiments. Representative images from one of the three independent experiments are shown. Statistical significance was determined by one-way ANOVA followed by Dunnett’s multiple comparisons test. ** *p* < 0.01 comparing 100% DM treatment to the untreated control (CTRL).

To evaluate the impact of DM on cell migration, an event closely associated with invasive potential and metastatic behavior, a wound healing assay on AGS cells was performed. As illustrated in Figure 4, treatment with 75% DM for 24 h led to a slight reduction in wound closure compared to untreated control cells, in which the scratch area was nearly completely repopulated. A significant (*p* = 0.0037) inhibition of migration was observed in cells treated with 100% DM for 24 h, suggesting a dose-dependent inhibitory effect of DM on cellular motility.

### 3.3. Cell Viability and Cytotoxicity Occurrence in the Presence of Donkey Milk: Dose-Dependent Effects

**Figure 5:**
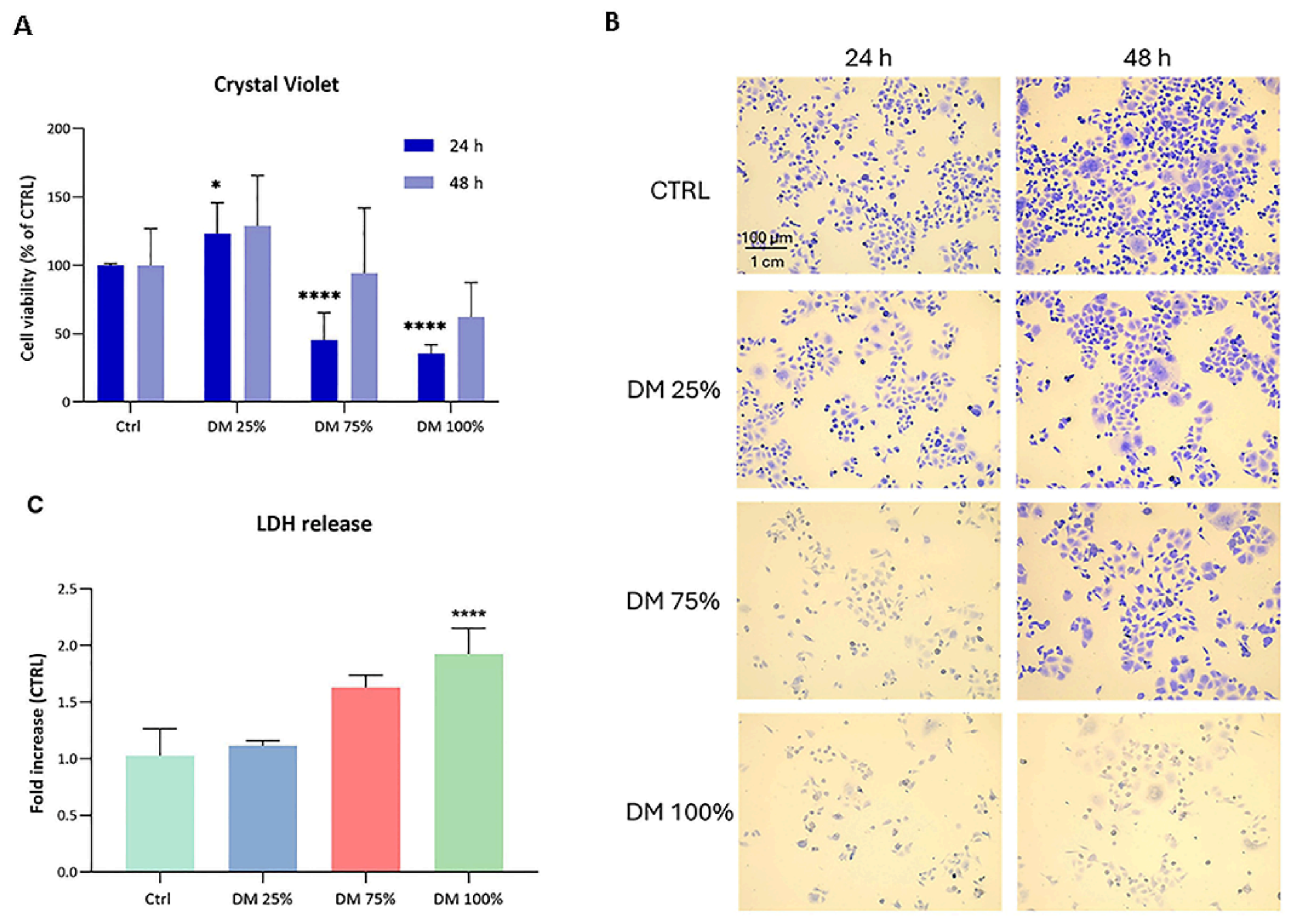
(**A**) Viability of AGS cells exposed to increasing concentrations of DM for 24 h and 48 h. The bar graphs represent cell viability percentages. The untreated control (CTRL) is set as 100%. Data are presented as means ± standard deviation obtained from one experiment in triplicate. * = *p* < 0.05, **** = *p* < 0.00001 comparing treated to the untreated control. (**B**) Images of crystal violet-stained AGS cells 24 h and 48 h after treatment. Magnification 10× (1 cm = 100 µm). (**C**) Cytotoxicity occurrence in AGS cells exposed to increasing concentrations of DM for 24 h. The bar graphs show the amount of lactate dehydrogenase (LDH) released from treated AGS cells as the fold increased from that secreted by untreated cells (CTRL) after 24 h of exposure. Data are presented as means ± standard deviation obtained from one experiment in triplicates. **** = *p* < 0.00001 comparing treated to the untreated control.

Cell viability, measured using the crystal violet assay after 24 and 48 h of exposure, is shown in Figure 5. After 24 h, AGS cells showed a slight, but significant increase in proliferation when 25% of DM was added to cultures. Cells exposed to 75% and 100% DM showed a decrease in viability in a dose-dependent manner at percentages lower than 50% versus control cells (Figure 5A). A similar trend was observed after 48 h of treatment. Regarding cell morphology, microscopic examination revealed that untreated AGS cells typically grew in dense clusters and exhibited a characteristic hexagonal shape, consistent with their epithelial origin. In contrast, following DM treatment, cluster density progressively decreased in a dose-dependent manner. Moreover, cells exhibited reduced staining and a more rounded morphology, indicative of reduced adherence and potential cytotoxic effects (Figure 5B). Data on cytotoxicity occurrence at 24 h (Figure 5C) confirmed those observed with the crystal violet assay: LDH release increased in a dose-dependent manner. The amount of release was approximately doubled with DM 100% compared to the untreated sample. Since 25% and 50% of DM displayed inefficacy on AGS cells, concentrations of 75% and 100% were used in subsequent experiments.

### 3.4. Induction of Mitochondrial Oxidative Stress

**Figure 6:**
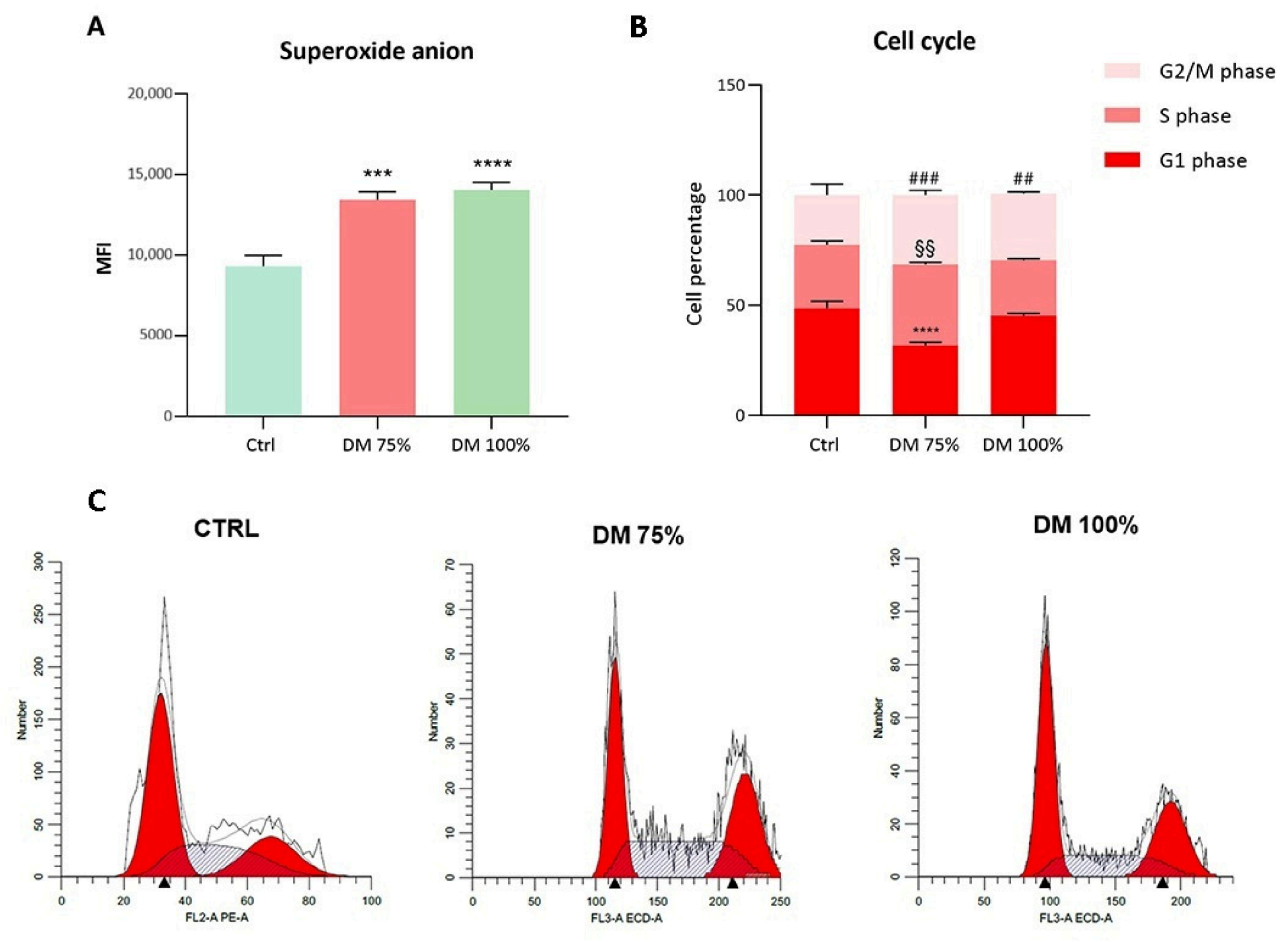
(**A**) Generation of superoxide anion in AGS cells exposed to increasing concentrations of DM after 24 h. The bar graph represents intracellular superoxide anions proportional to the MFI (mean fluorescence intensity) in the phycoerythrin channel (FL-2). *** *p* < 0.0001 and **** *p* < 0.00001 comparing treated to the untreated control. (**B**) Cell cycle analysis in AGS cells exposed to increasing concentrations of DM after 48 h. Data are presented as means ± standard deviation from three independent experiments. Bars highlight cell percentages in the various phases of the cell cycle (G1, S, and G2/M) of AGS. **** *p* <0.00001 comparing treated to the untreated control (G1 phase). §§ *p* < 0.001 comparing treated to the untreated control (S phase). ## *p* < 0.001, ### *p* < 0.0001 comparing treated to the untreated control (G2/M phase). (**C**) The panel displays the DNA profile of cells 48 h after treatment. Peaks are generated by the emission of PI in the FL3 fluorescence channel.

To investigate whether the observed reduction in cell viability and proliferation, along with the induction of cytotoxicity, could be associated with oxidative stress, mitochondrial superoxide production was assessed by flow cytometry after 24 h of exposure to selected concentrations of DM (Figure 6A). The results revealed a dose-dependent increase in mitochondrial superoxide anion levels, suggesting potential involvement of oxidative mechanisms in DM-induced cellular effects.

### 3.5. Analysis of Cell Cycle Progression

Percentages of cells in the various phases of the cell cycle were measured by flow cytometry after 48 h (Figure 6B,C) to confirm crystal violet assay results and to investigate in which checkpoint AGS cells arrest their proliferation. The administration of 75% DM resulted in a significant decrease in the proportion of cells in the G1 phase, along with an increase in both the S and G2 phases, compared with untreated controls. Conversely, when cells were exposed to 100% DM, the cell cycle profile was comparable to that of the untreated controls, although a slight but statistically significant increase in the G2 phase was observed (*p* < 0.001).

### 3.6. Donkey Milk Modulates Inflammatory Cytokine Expression in AGS Cells

**Figure 7:**
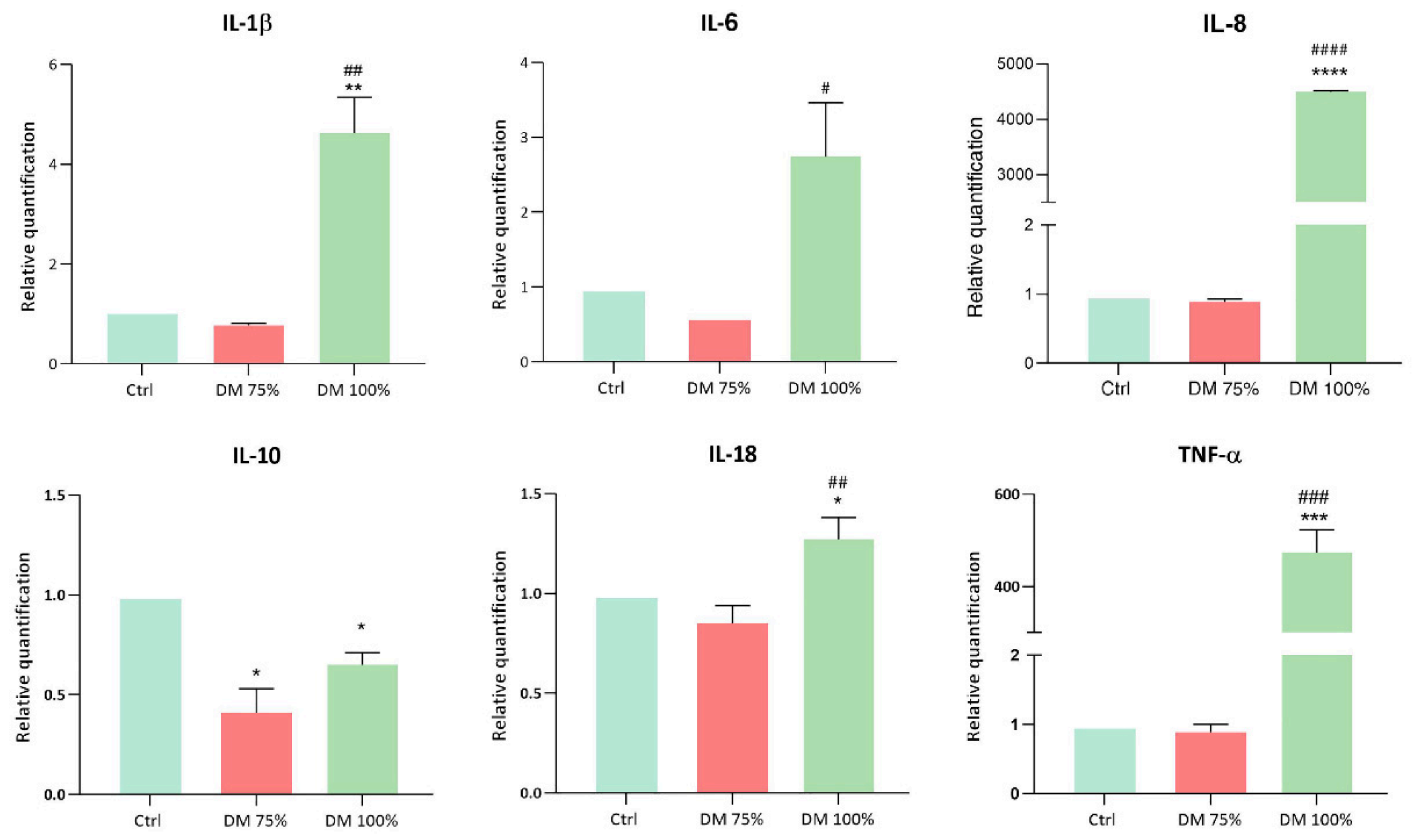
Effects of 75% DM in complete medium and 100% DM on IL-1β, IL-6, IL-8, IL-10, IL-18 and TNF-α relative gene expression after 24 h treatment in AGS cell line determined by real-time RT PCR. Data were calculated using the 2^−ΔΔCt^ method, normalized to β-actin cDNA levels and then expressed as relative to control (calibrator sample, defined as 1.00). Data are expressed as means ± SD and were analyzed by analysis of variance (ANOVA) followed by Tukey’s multiple comparison test. * *p* < 0.05, ** *p* < 0.01, *** *p* < 0.005, **** *p* < 0.001 comparing treated to the untreated control. ^#^ *p* < 0.05, ^##^ *p* < 0.01, ^###^ *p* < 0.005, ^####^ *p* < 0.001 comparing 100% DM treatment to the 75% DM treatment.

The expression profile of a panel of pro- and anti-inflammatory cytokines was analyzed in AGS cells following treatment. The mRNA levels of IL-1β, IL-6, IL-8, IL-10, IL-12, IL-18, and TNF-α were quantified by qRT-PCR after 24 h of exposure to 75% and 100% DM, in order to evaluate the potential immunomodulatory effects of DM at the molecular level. As shown in Figure 7, IL-1β gene expression was moderately increased after 24 h of treatment with 100% DM compared to both 75% DM and untreated cells (*p* < 0.01). A slight but significant upregulation of IL-6 was also observed under 100% DM treatment compared to the 75% DM (*p* < 0.05). Notably, a marked upregulation of IL-8 and TNF-α expression levels was detected following 100% DM exposure, with statistically highly significant differences compared to all other conditions (*p* < 0.001). In contrast, the expression levels of IL-10 and IL-18 were reduced in both DM-treated cells compared to control cells, with the exception of IL-18, which showed a slight but significant increase in response to 100% DM. IL-12 gene expression was undetectable under all tested conditions.

### 3.7. Donkey Milk Modulates Non-Apoptotic Cell Death in AGS Cell Line

**Figure 8:**
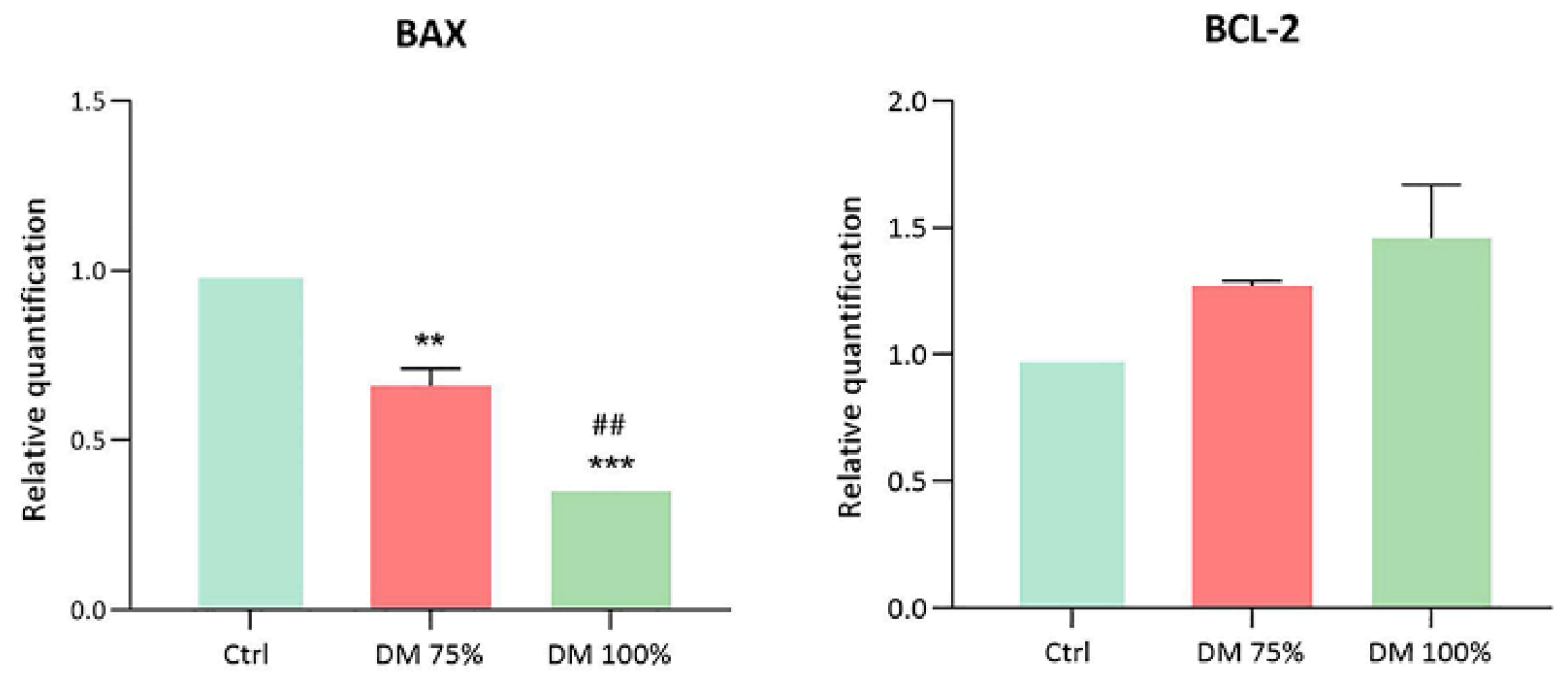
Effects of 75% DM in complete medium and 100% DM on BCL-2 and BAX relative gene expression after 24 h treatment in AGS cell line determined by real-time RT PCR. Data were calculated using the 2^−ΔΔCt^ method, normalized to β-actin cDNA levels and then expressed as relative to control (calibrator sample, defined as 1.00). Data are expressed as means ± SD and were analyzed by analysis of variance (ANOVA) followed by Tukey’s multiple comparison test. ** *p* < 0.01, *** *p* < 0.005 comparing treated to the untreated control. ## *p* < 0.01 comparing 100% DM treatment to the 75% DM treatment.

Due to the persistent milk-derived particulate interference, a direct assessment of apoptotic events by FACS was not feasible. As an alternative approach, gene expression profiling was employed to investigate the potential involvement of apoptotic pathways in DM-induced cytotoxicity. The expression of two key regulators of the mitochondrial apoptotic pathway, BAX (pro-apoptotic) and BCL-2 (anti-apoptotic), was evaluated using qRT-PCR (Figure 8). The relative expression of these genes is commonly used to infer apoptotic commitment at the molecular level. BAX expression levels were moderately reduced following 24 h exposure to both 75% and 100% DM (*p* < 0.01 and *p* < 0.005 respectively) in a dose-dependent manner compared to untreated cells. Conversely, although BCL-2 expression levels showed a slight dose-dependent increase under the same conditions (*p* > 0.05), this difference did not reach statistical significance (*p* > 0.05).

## 4. Discussion

Gastric cancer (GC) is a major global oncology challenge, ranking fifth in incidence and fourth in mortality worldwide. Adenocarcinomas constitute ~95% of cases, showing significant geographic, ethnic, and gender disparities. Because early-stage GC is frequently asymptomatic or presents with non-specific symptoms, delayed diagnosis is common, causing 5-year survival rates to drop from ~75% in localized disease to under 10% in metastatic cases [2,3,4,42]. While upper endoscopy with biopsy remains the diagnostic gold standard, prognosis depends on tumor stage, histological differentiation, anatomical location, and molecular biomarkers like HER2, MSI, and PD-L1 [42,43,44]. Despite preventive strategies—such as *H. pylori* eradication and national screening programs—improving early detection, most cases are still diagnosed at an advanced stage [8,9,10]. Surgery is the cornerstone of curative treatment, combined with chemotherapy, chemoradiotherapy, targeted therapy, and immunotherapy, particularly in biomarker-selected patients [6,45]. However, therapeutic response and resistance are heavily complicated by tumor heterogeneity, the tumor microenvironment, and gastric cancer stem cells [5,6,7,46]. In this scenario, non-toxic, nutritionally derived adjunctive therapies are gaining attention. Donkey milk (DM) is of particular interest due to its similarity to human milk (HM) in protein composition and bioactive compounds, alongside its established hypoallergenic properties and pediatric nutritional benefits [11,12,13,14]. Immunologically, DM exerts context-dependent modulatory effects, enhancing immune responsiveness in elderly individuals and showing anti-inflammatory activity in metabolic and intestinal disorders [47,48,49,50,51]. Although preclinical models support its gut-protective and antioxidant effects, the underlying molecular mechanisms remain incompletely defined [52,53,54,55]. Values observed in DM used in the present study were in line with values reported in the literature [37] and confirmed protein and lactose contents close to that of HM while highlighting the extremely low lipid (and therefore energy) content. The ash content of DM was intermediate between HM and cow milk. Regarding nitrogenous content, urea concentration was lower in DM than HM, reflecting species-specific recycling abilities. The compositional stability across lactation stages allowed the use of pooled bulk milk for all in vitro assays, ensuring reproducibility. The observed inhibition of cell migration and reduction in cell viability at higher DM concentrations addressed the research question regarding the potential anticancer properties of DM in GC. The data suggest that DM limits the proliferative and migratory capacity of AGS cells and may induce cytotoxicity in a dose-dependent manner associated with oxidative stress and cell cycle disruption. Interestingly, the three experimental outcomes (i.e., cell viability, LDH release and wound closure) displayed different sensitivity thresholds to DM treatment. Already at the sub-lethal concentration of 75% DM, cell viability was significantly reduced compared to untreated controls, as measured by crystal violet staining. However, this reduction was not accompanied by a significant increase in LDH release, indicating that overt membrane damage and cytotoxic cell death were not yet occurring at this concentration. Likewise, directional cell migration was not significantly affected at 75% DM, despite the parallel reduction in cell viability. Only at 100% DM did both LDH release and inhibition of wound closure reach statistical significance compared to control, occurring concomitantly. This dose-response pattern indicates that a reduction in cell viability/proliferation is the earliest and most sensitive read-out of DM exposure, while overt cytotoxicity (as reflected by LDH release) and impairment of cell motility emerge together only at the highest concentration tested, suggesting that the anti-migratory effect of DM may be more closely linked to the onset of cytotoxic damage than to an early, cytotoxicity-independent inhibition of motility pathways. These findings are consistent with in vivo evidence from 4T1 triple-negative breast cancer (TNBC), where both donkey colostrum (DC) and mature milk (DM) are shown to suppress tumor metastasis and vascular development. Specifically, treatment with DC or mature DM led to a significant downregulation of MMP-2, a key marker of metastatic activity, and CD31, a key player in tumor-induced vascularization. This suggests that DM and DC may inhibit metastasis to secondary organs by reducing MMP-2 expression in tumor cells and limiting vascular infiltration within the tumor microenvironment. Additionally, analysis of cell adhesion markers revealed that DM upregulated E-cadherin expression, a molecule typically associated with reduced invasiveness and enhanced cell–cell adhesion, while also increasing the number of N-cadherin–expressing cells in tumor tissues. Although DC also increased E-cadherin expression, this effect did not reach statistical significance compared to controls [36]. This DM-induced remodeling of cell adhesion proteins strongly correlates with the morphological alterations and the reduction in cell adherence observed in our in vitro assays, providing a robust molecular rationale for the anti-migratory and anti-proliferative effects of DM observed at the highest concentration tested.

The dose-dependent reduction in cell viability observed following treatment with 75% and 100% DM at both 24 and 48 h is consistent with previous studies reporting decreased viability in cancer cells treated with DM or its components. Esener et al. [35] reported that DM kefir induced apoptosis and suppressed proliferation in Ehrlich ascites carcinoma in mice. Apoptotic effects were evaluated through immunohistochemical staining using the histological H-score method [35]. Via MTT assay, Mao et al. [56] demonstrated that the active fraction of DM significantly reduced the viability of A549 lung carcinoma cells in a dose- and time-dependent manner. Similarly, Shariatikia et al. [34] observed strong cytotoxic activity against MCF-7 breast cancer cells in a dose-dependent fashion exerted by both the casein and whey protein fractions of DM. Consistently, Wang et al. [57] investigated the effects of donkey milk oligosaccharides (DMOs) on HT-29, Caco-2, and HIEC intestinal cell lines. While exposure time had minimal impact, a clear concentration-dependent decrease in proliferation was observed across all three lines following DMOs treatment.

In contrast, Li et al. [58] reported that DM exerted a protective effect on damaged MIN6 cells, a mouse pancreatic beta-cell line. In this case, cell viability increased in a dose-dependent manner across all treatment groups, highlighting the potential regenerative or cytoprotective properties of DM under specific conditions. The promotion of regenerative processes was supported by Kocic et al. [59]: in their study on skin fibroblast DM, especially its non-casein bioactive peptides, promoted regenerative processes by activating the Erk pathway in skin fibroblasts. The results of the present study revealed that treatment with 75% DM significantly altered the cell cycle distribution in AGS cells, with a reduction in the G1 phase and an increase in the S and G2 phases, suggesting potential impaired proliferation upon cytotoxicity. Conversely, 100% DM treatment induced a modest, but significant accumulation in the G2 phase, possibly reflecting a checkpoint block associated with oxidative stress and reduced metabolic activity. This divergence suggests that DM may exert context-specific cytotoxic effects, which in AGS cells do not rely on classical caspase-dependent apoptosis, but may involve alternative forms of regulated cell death.

These findings show partial concordance with previous studies. Mao et al. [56] demonstrated that DM active fraction arrested A549 lung cancer cells at the G0/G1 and G2/M phases, with a concurrent induction of apoptosis. Similarly, our 75% DM-treated cells exhibited an S/G2 accumulation, suggesting a comparable antiproliferative action via disruption of cell cycle progression, though the phase distribution differs likely due to cell type-specific responses or the use of whole DM versus isolated fractions. Additionally, Wang et al. [57] investigated DMOs and reported dose-dependent cell cycle arrest in intestinal cell lines: HT-29 and Caco-2 cells showed G2/M arrest at higher DMO concentrations, associated with downregulation of CDC2 and cyclin B1 and activation of the p38 MAPK pathway. Although cyclin expression or signaling pathways was not assessed in the present study, the observed G2 phase accumulation and ROS increase in the 100% DM group was consistent with stress-induced checkpoint activation, possibly involving similar mechanisms [57]. These findings add to a growing body of evidence that DM or its components exert antiproliferative activity across multiple cancer cell types. However, the phase of cell cycle arrest appears to be model-dependent, suggesting that distinct tumor types may respond differently to DM-induced growth inhibition.

The unexpected pro-inflammatory gene response observed in AGS cells following DM treatment highlights the context-dependent nature of DM immunomodulatory effects. While DM is known for its anti-inflammatory properties in healthy or inflamed tissues, its impact on tumor cells may differ, potentially reflecting a stress response or a shift toward cell death pathways. The pro-inflammatory gene induction observed in AGS cells may reflect an immunostimulatory property of DM that, while beneficial in immune-compromised or aged subjects, could instead activate stress-related pathways in tumor cells.

As described above, DM demonstrates a complex immunological interaction that varies with age and physiological context. Tafaro et al. [47], in an in vitro study, demonstrated that DM from the Martina Franca breed potently activated human peripheral blood mononuclear cells (PBMCs). Specifically, mature milk significantly increased IgG production, while colostrum predominantly stimulated IgA as a key for mucosal immunity, upregulated activation markers (CD25/CD69) and induced the secretion of both pro-inflammatory cytokines (IL-1β, IL-12, TNF-α) and anti-inflammatory mediators (IL-10), along with nitric oxide release [47].

These findings were consolidated in a subsequent review by Jirillo et al. [49], who emphasized the ability to concurrently stimulate IL-12, TNF-α, IL-10, and nitric oxide in PBMCs, suggesting a dual mechanism of immune activation and homeostatic regulation.

Interestingly, the immunostimulatory effects of DM appear to be more pronounced in elderly individuals. In a clinical study, Amati et al. [48] reported that one-month daily consumption of DM significantly increased serum levels of IL-1β, IL-6, IL-8, and TNF-α in elderly subjects, indicating enhanced immune responsiveness and aligning with our results. Intriguingly, the same clinical study reported undetectable IL-12 and only minimal changes in IL-10 serum levels, which is consistent with our in vitro findings. Similarly, another in vitro study demonstrated elevated levels of TNF-α, IL-6, IL-8, and IL-10 in PBMCs; however, a reduction in IL-10 levels in aged donors suggested impaired regulatory feedback and confirmed the age-associated decline in regulatory cytokine production [51]. These results were further supported by Mao et al. [56], who showed that active fractions of DM could stimulate the production of IL-2, IFN-γ, IL-6, TNF-α, and IL-1β by splenocytes and macrophages in vitro. Our findings significantly strengthen this line of evidence, demonstrating a clear and coordinated upregulation of key pro-inflammatory mediators (IL-1β, IL-6, IL-8, and TNF-α) after exposure to 100% DM. Crucially, this robust activation of the acute phase response is further substantiated by the concurrent and significant downregulation of IL-10 gene expression observed in our DM-treated cells. Given that IL-10 is a pivotal anti-inflammatory cytokine responsible for dampening immune reactivity and maintaining immune homeostasis, its suppression strongly argues against a simple passive state. Instead, it indicates that DM actively reprograms the cellular cytokine profile to favor a transient, alert immune status. This apparent paradox—where a substance traditionally renowned for its anti-inflammatory benefits triggers a pro-inflammatory signaling cascade—can be explained by the biological role of milk as a physiological primer. Rather than inducing chronic or detrimental inflammation, DM appears to act as a potent immunomodulator capable of “boosting” or priming the innate immune system. The induction of early response cytokines (like TNF-α and IL-1β) combined with the silencing of the IL-10 brake suggests that DM provides a crucial stimulatory signal. This mechanism could be evolutionary designed to enhance host defense mechanisms, preparing the organism (particularly newborns or immunodeficient/elderly individuals) to mount a faster and more effective response against potential pathogens. Conversely, rodent studies have yielded differing results. Trinchese et al. [50] reported systemic anti-inflammatory effects of DM, including reductions in pro-inflammatory cytokines (TNF-α, IL-1) and endotoxin (LPS) levels, along with increased IL-10. A significant reduction in these same cytokines, as well as in IL-6 and monocyte chemoattractant protein 1 (MCP-1), was also reported in a novel study investigating the effects and mechanisms of DM supplementation in chronic kidney disease, specifically focusing on its anti-inflammatory properties and its impact on renal fibrosis in a rat model [60]. In our study, the immunomodulatory and apoptotic profiles of DM-treated AGS cells were characterized comprehensively at the transcriptional level via qRT-PCR. It is worth noting that while validating mRNA expression profiles with protein-level analyses (such as Western blotting or ELISA) represents a standard approach, the evaluation of whole, complex food matrices like donkey milk poses unique and significant technical challenges in vitro. Whole DM is naturally characterized by an extremely high abundance of native milk proteins (e.g., caseins, lysozyme, and lactoferrin) and endogenous immunoglobulins. In our experimental setting, the persistence of these dense matrix components within the cell microenvironment introduces severe molecular interference, high non-specific background noise, and antibody cross-reactivity, which ultimately compromised the reliability and reproducibility of standard protein assays on cell lysates or supernatants. Consequently, gene expression profiling remained the most precise, robust, and interference-free methodology to elucidate the specific molecular pathways activated by DM. Importantly, the dramatic transcriptional upregulation of pro-inflammatory and acute-phase markers (such as IL-1β, IL-6, IL-8, and TNF-α) strongly and functionally correlates with the macroscopic cytotoxicity, cell rounding, and extensive LDH release observed in our phenotypic assays, confirming that the documented mRNA alterations directly mirror the severe biological disruption of gastric cancer cells.

Importantly, the absence of classical apoptotic signaling in the present study suggests that DM-induced cytotoxicity in AGS cells may be mediated through alternative, non-apoptotic mechanisms. This contrasts with previous reports indicating pro-apoptotic effects of DM or its derivatives in other cancer models. For instance, Li et al. [36] demonstrated increased Bax expression in TNBC tissues following treatment with DC and mature DM, indicating enhanced apoptosis via the intrinsic pathway. Similarly, Esener et al. [35] demonstrated apoptosis promotion by active caspase 3 staining and TUNEL assay. The apparent divergence in cell death pathways highlights the complexity of DM biological activity and opens new avenues for research into non-canonical mechanisms of cell death. Elucidating these alternative pathways may provide critical insights into how DM could be harnessed as an adjunctive therapeutic agent, particularly in targeting tumor cells resistant to apoptosis. However, interpretation of the present study results should take into account some limitations. First, the whole DM without fractionation was used, which reflects real-world use, but limits mechanistic insights into specific bioactive components. Second, the experiments were conducted only in the AGS cell line, which, despite being widely used, cannot capture the heterogeneity of GC or the complexity of tumor–microenvironment interactions. Third, while endpoints such as proliferation, migration, oxidative stress, and gene expression were assessed, deeper mechanistic analyses and direct comparisons with other milk matrices were not performed. Fourth, due to limited sample availability and resource constraints, analysis was restricted to the transcriptional level. Consequently, whether the observed gene expression changes are reflected at the protein level remains to be validated via Western blot or other proteomic approaches. Finally, the absence of validation in animal or organoid models restricts translational applicability, warranting further in vivo and preclinical studies.

## 5. Conclusions

This study demonstrates that DM inhibits cell migration, reduces cell viability, induces oxidative stress, alters cell cycle progression, and modulates inflammatory responses in AGS cells. In particular, treatment with high DM concentrations (75% and 100%) resulted in significant reductions in cell viability and migratory capacity, accompanied by increased LDH release and mitochondrial superoxide production. Moreover, DM exposure promoted cell cycle alterations characterized by S/G2 phase accumulation and enhanced expression of pro-inflammatory mediators. Its bioactivity appears context-dependent and is consistent with non-apoptotic cytotoxic mechanisms. These results support further investigation of DM as a nutraceutical adjunct in GC, including studies in organoid and animal models, isolation of active components, and potential clinical applications. The use of traceable, sustainable DM highlights the feasibility of integrating agricultural sourcing with biomedical research for translational purposes.

## Figures and Tables

**Table 1:** Primer sequences for target genes.

Gene	Forward Primer (5′–3′)	Reverse Primer (5′–3′)
IL-1β	TGAGGATGACTTGTTCTTTGAAG	GTGGTGGTCGGAGATTCG
IL-6	GTACATCCTCGACGGCATC	ACCTCAAACTCCAAAAGACCAG
IL-8	GTGTAAACATGACTTCCAAGCT	GTCCACTCTCAATCACTCTCAG
IL-10	GAGAACCAAGACCCAGACATC	TCACTCATGGCTTTGTAGATGC
IL-12	CGGTTCACAAGCTCAAGTATG	GCAGAATGTCAGGGAGAAGTAG
IL-18	CAGTCAGCAAGGAATTGTCTC	GAGGAAGCGATCTCGGAAGG
TNF-α	CCTTCCTGATCGTGGCAG	GCTTGAGGGTTTGCTTACAAC
Bax	GCCCTTTTGCTTCAGGGTTTC	CATCCTCTGCAGCTCCATGT
BCL-2	GTGTGGAGAGCGTCAACC	TACCCAGCCTCCGTTATC

## Data Availability

All data are included in the manuscript. Additional information can be provided by the corresponding author based on reasonable request.
